# GYNOCARE Update: Modern Strategies to Improve Diagnosis and Treatment of Rare Gynecologic Tumors—Current Challenges and Future Directions

**DOI:** 10.3390/cancers13030493

**Published:** 2021-01-27

**Authors:** Riccardo Di Fiore, Sherif Suleiman, Bridget Ellul, Sharon A. O’Toole, Charles Savona-Ventura, Ana Felix, Valerio Napolioni, Neil T. Conlon, Ilker Kahramanoglu, Miriam J. Azzopardi, Miriam Dalmas, Neville Calleja, Mark R. Brincat, Yves Muscat-Baron, Maja Sabol, Vera Dimitrievska, Angel Yordanov, Mariela Vasileva-Slaveva, Kristelle von Brockdorff, Rachel A. Micallef, Paul Kubelac, Patriciu Achimas-Cadariu, Catalin Vlad, Olga Tzortzatou, Robert Poka, Antonio Giordano, Alex Felice, Nicholas Reed, C. Simon Herrington, David Faraggi, Jean Calleja-Agius

**Affiliations:** 1 Department of Anatomy, Faculty of Medicine and Surgery, University of Malta, MSD 2080 Msida, Malta; riccardo.difiore@um.edu.mt (R.D.F.); sherif.s.suleiman@um.edu.mt (S.S.); 2 Center for Biotechnology, Sbarro Institute for Cancer Research and Molecular Medicine, College of Science and Technology, Temple University, Philadelphia, PA 19122, USA; president@shro.org; 3 Centre for Molecular Medicine & Biobanking, University of Malta, MSD 2080 Msida, Malta; bridget.ellul@um.edu.mt; 4 Departments of Obstetrics and Gynaecology and Histopathology, Trinity St James’s Cancer Institute, Trinity College Dublin, Dublin 8, Ireland; shotoole@tcd.ie; 5 Department of Obstetrics and Gynaecology, Faculty of Medicine and Surgery, University of Malta, MSD 2080 Msida, Malta; charles.savona-ventura@um.edu.mt; 6 Department of Pathology, Campo dos Mártires da Pátria, Instituto Portugues de Oncologia de Lisboa, NOVA Medical School, UNL, 130, 1169-056 Lisboa, Portugal; ana.felix@nms.unl.pt; 7 Genomic And Molecular Epidemiology (GAME) Lab., School of Biosciences and Veterinary Medicine, University of Camerino, 62032 Camerino, Italy; valerio.napolioni@unicam.it; 8 National Institute for Cellular Biotechnology, Dublin City University, Glasnevin, 9 Dublin, Ireland; neil.conlon@dcu.ie; 9 Department of Gynecologic Oncology, Emsey Hospital, Istanbul 3400, Turkey; ilker.kahramanoglu@emseyhospital.com; 10 Directorate for Health Information and Research, PTA 1313 G’Mangia, Malta; miriam.j.azzopardi@gov.mt (M.J.A.); neville.calleja@gov.mt (N.C.); 11 Office of the Chief Medical Officer, Department of Policy in Health, Ministry for Health, 15 Merchants Street, VLT 1171 Valletta, Malta; miriam.dalmas@gov.mt; 12 Department of Obstetrics and Gynaecology, Mater Dei Hospital, Triq Dun Karm, MSD 2090 Msida, Malta; M.brincat1@nhs.net (M.R.B.); yambaron2018@gmail.com (Y.M.-B.); 13 Laboratory for Hereditary Cancer, Division of Molecular Medicine, Ruđer Bošković Institute, 10000 Zagreb, Croatia; maja.sabol@irb.hr; 14 University of American College, 1000 Skopje, North Macedonia; vdimitrievska@gmail.com; 15 Department of Gynecologic Oncology, Medical University Pleven, 5800 Pleven, Bulgaria; angel.jordanov@gmail.com; 16 Department of Surgery, Alexandrovska University Hospital, 1431 Sofia, Bulgaria; sscvasileva@gmail.com; 17 Sir Anthony Mamo Oncology Centre, Department of Oncology and Radiotherapy, Mater Dei Hospital, MSD 2090 Msida, Malta; kristel-marie.von-brockdorff@gov.mt (K.v.B.); rachel.a.micallef@gov.mt (R.A.M.); 18 Department of Medical Oncology, The Oncology Institute “Prof. Dr. Ion Chiricuţă”. 34–36 Republicii Street, 400015 Cluj-Napoca, Romania; kubelac.paul@umfcluj.ro; 19 Department of Oncology, “Iuliu Hatieganu” University of Medicine and Pharmacy, 400012 Cluj-Napoca, Romania; pachimas@umfcluj.ro (P.A.-C.); catalin.vlad@umfcluj.ro (C.V.); 20 Department of Surgical Oncology, The Oncology Institute “Prof. Dr. Ion Chiricuta”, 400015 Cluj-Napoca, Romania; 21 Department of Surgery, The Oncology Institute “Prof. Dr. Ion Chiricuta”, 400015 Cluj Napoca, Romania; 22 Biomedical Research Foundation of the Academy of Athens, Soranou Efesiou 4 str., 11527 Athens, Greece; otzortzatou@bioacademy.gr; 23 Institute of Obstetrics and Gynaecology, University of Debrecen, Nagyerdei krt. 98, 4032 Debrecen, Hungary; pokar@med.unideb.hu; 24 Department of Medical Biotechnologies, University of Siena, 53100 Siena, Italy; 25 Centre of Molecular Medicine and BioBanking, Department of Physiology & Biochemistry, Faculty of Medicine & Surgery, University of Malta, MSD 2080 Msida, Malta; alexfelice1@icloud.com; 26 Beatson Oncology Centre, Gartnavel General Hospital, 1053 Great Western Road, Glasgow G12 0YN, UK; Nick.Reed@ggc.scot.nhs.uk; 27 Cancer Research UK Edinburgh Centre, Western General Hospital, University of Edinburgh, Crewe Road South, Edinburgh EH4 2XR, UK; Simon.herrington@ed.ac.uk; 28 Department of Statistics, University of Haifa, Haifa 31905, Israel; dfaraggi@univ.haifa.ac.il

**Keywords:** rare gynecologic tumors, circulating tumor-specific markers, cancer stem cells, theranostics, biobanking, personalized medicine

## Abstract

**Simple Summary:**

More than 50% of all the tumors affecting the female genital tract can be classified as rare and usually have a poor prognosis owing to delayed diagnosis and treatment. Currently, gynecologic cancer research, due to distinct scientific and technological challenges, is lagging behind. Moreover, the overall efforts for addressing these challenges are fragmented across different countries. The European Network for Gynecological Rare Cancer Research: GYNOCARE aims to address these challenges by creating a unique network between key stakeholders covering distinct domains from basic research to cure. GYNOCARE is part of a European Collaboration in Science and Technology (COST) with the aim to focus on the development of new approaches to improve the diagnosis and treatment of rare gynecological tumors. Here, we provide a brief overview describing the goals of this COST Action and its future challenges with the aim to continue fighting against this rare cancer.

**Abstract:**

More than 50% of all gynecologic tumors can be classified as rare (defined as an incidence of ≤6 per 100,000 women) and usually have a poor prognosis owing to delayed diagnosis and treatment. In contrast to almost all other common solid tumors, the treatment of rare gynecologic tumors (RGT) is often based on expert opinion, retrospective studies, or extrapolation from other tumor sites with similar histology, leading to difficulty in developing guidelines for clinical practice. Currently, gynecologic cancer research, due to distinct scientific and technological challenges, is lagging behind. Moreover, the overall efforts for addressing these challenges are fragmented across different European countries and indeed, worldwide. The GYNOCARE, COST Action CA18117 (European Network for Gynecological Rare Cancer Research) programme aims to address these challenges through the creation of a unique network between key stakeholders covering distinct domains from concept to cure: basic research on RGT, biobanking, bridging with industry, and setting up the legal and regulatory requirements for international innovative clinical trials. On this basis, members of this COST Action, (Working Group 1, “Basic and Translational Research on Rare Gynecological Cancer”) have decided to focus their future efforts on the development of new approaches to improve the diagnosis and treatment of RGT. Here, we provide a brief overview of the current state-of-the-art and describe the goals of this COST Action and its future challenges with the aim to stimulate discussion and promote synergy across scientists engaged in the fight against this rare cancer worldwide.

## 1. Introduction

It is estimated that, globally, gynecologic malignancies comprise 19% of the new cancer diagnoses in women [1]. Up to 50% of these tumors are classified as rare (Table 1) [2,3]. Any strategy to improve on the available knowledge of rare gynecological malignancies requires a clear definition of what conditions are considered rare. The term ‘rare tumor’ refers mostly to non-epithelial subtypes. However, histologically different epithelial subtypes of ovarian, endometrial and cervical cancers are also to be categorized as rare tumors (with an annual incidence of six or fewer per 100,000) due to their different pathological behaviors [4]. In total, rare gynecologic tumors (RGT) represent more than 50% of the total number of gynecologic tumors, with approximately 80,000 new cases per year in Europe. In addition, these involve more than 30 different histologic diagnoses, with a very limited number of patients in each diagnostic category [3]. This is in stark contrast with other common solid tumors. RGT are also associated with a poor prognosis, mainly because of delayed diagnosis due to clinical inexperience, the lack of knowledge and limited therapeutic options [1].

Currently, the treatment of RGT is often based on retrospective studies, expert opinion, and/or extrapolation from other tumor sites with similar histology. This leads to difficulty in developing guidelines for clinical practice [1,5,6,7]. Hence, the management of these tumors needs to be based on scientific evidence that should lead to international consensus guidelines and clinical trials, as well as reference centers and/or networks sharing multidisciplinary expertise and access to clinical trials [8,9]. Many of the difficulties in conducting trials in RGT subtypes could be overcome through the establishment of robust international collaborations [9]. The establishment of networks of centers for RGT across the European Union (EU) helps to achieve the necessary organizational structure and critical mass to improve the biological knowledge of these diseases, carry out clinical trials, and thus optimise patient care [9]. In this regard, the European Commission is implementing the Directive 2011/24/EU that is meant to grant EU patients the right to access safe and good-quality treatment across EU borders. In particular, the creation of the European Reference Networks (ERNs) intends to provide specialised healthcare for rare diseases [10]. The formal activation of ERNs is a cornerstone in the EU cooperation on rare cancers, along with the established Joint Action on Rare Cancers (JARC) of the European Public Health Programme [10]. Indeed, JARC, launched in October 2016, was a 3-year initiative aimed to optimize the creation process of the ERNs [11].

Since March 2017, the 24 existing ERNs for rare diseases are serving as research and knowledge centers, with the scope of treating patients from other EU Member States, and updating and contributing to the latest scientific findings. In 2016, EURACAN (Rare Adult CANcer) was set up to develop an EU network dedicated to rare adult cancers (RAC), with the aim to establish a world-leading, patient-centric and sustainable network of multidisciplinary research-intensive clinical center. The aim is to standardize and improve the quality of care of all RAC in European adult patients and ensure an optimised access to clinical innovation in the field of RAC and across all EU Member States. In association with patients advocacy groups, multilanguage information documents will be prepared, specifically outlining the nature of the disease, treatments, management, and reference centres for treatment and support.

**Figure 1:**
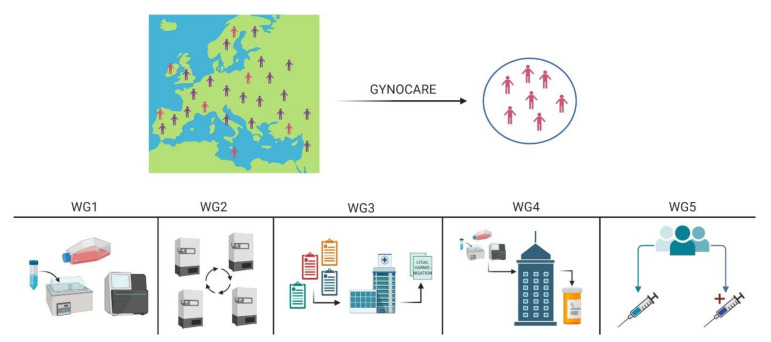
Description of the GYNOCARE COST Action CA18117. The main aim and objective of the Action is to create a European platform for Gynaecological Rare Cancer research to bridge the gap from concept to cure (connect basic research to biobanking to clinical trials). GYNOCARE has devised 5 Working Groups (WGs) and each of which contributes to a specific sub-objective and tackles a specific challenge. WG1–Basic Research: Coordination of ongoing and future research activities. The main objective of WG1 is to further develop a well-established network of researchers that impulse research in Gynaecological Cancer, with focus on very rare diseases where the treatments options are scarce. WG2–Coordination of bridging the gap between biobanks and translational research projects: The main objectives of WG2 are to establish a virtual network from the existing European biobanks for rare gynaecological malignancies (using a virtual platform that will allow the real time visualisation of the samples); and to integrate the biobanking concept within the clinical trials and translational research projects running in this field. WG3–Coordinating harmonisation of legal/regulatory requirements for international trials and other collaborative efforts. This WG aims to harmonise the legal requirements requested from the different European countries (all EU countries and non-EU countries within Europe). WG4-Bridging the gap between industry and biotechnology companies and translational research projects. The main objective of WP4 is to introduce GYNOCARE to the Pharma industries and to the companies that are developing commercial tools for diagnosis and prognostic assessment of the patients to showcase the distinct value for trial and study design, while also revealing to potential for smarter drug design and reuse of existing therapeutics. WG5–Coordination of interactions between clinical trials, translational research, and basic research. The main objective of the WP is to connect all the actions and stakeholders to existing and established clinical trial activities. (https://www.cost.eu/actions/CA18117/#tabs|Name:overview). Figure created using Biorender (https://biorender.com/).

However, at present, gynecologic cancer research, due to distinct scientific and technological challenges, is lagging. GYNOCARE, COST Action CA18117 (European Network for Gynecological Rare Cancer Research) is an EU funded programme that aims to address these challenges by creating a unique network between key stakeholders covering distinct RGT research areas ranging from concept to cure basic research, biobanking, bridging with industry, and setting up the legal and regulatory requirements for international innovative clinical trials (Figure 1) [12]. On this basis, members of this COST Action, (Working Group 1, “Basic and Translational Research on Rare Gynecological Cancer”) have decided to focus their future efforts on the development of new approaches to improve the diagnosis and treatment of RGT. The future challenges will be described, and the action which will be launched in order to achieve ambitious goals, taking into account both the state-of-the-art and vision of this COST Action. Getting the histopathology right, with centralised referrals and review of pathology, which is now readily available with digital image transfer. Correct histopathological diagnosis leads to correct treatment and potentially better outcome. This is nowadays simple technology, which, with endorsement, can be implemented quickly and cheaply.

## 2. Challenges

### 2.1. Definition of Rare Gynaecological Tumors

The primary challenge is definition of what truly constitutes a rare gynaecological tumor. Apart from the updated classification of rare gynecological malignancies based on site and morphological criteria as given in Table 1, the European Society of Gynae-Oncology (ESGO) has recently launched a mobile app as part of the rare cancers algorithms and guidelines [13]. Leiomyosarcoma, carcinosarcoma, malignant sex cord-stromal tumor, malignant germ cell tumor, gestational trophoblastic disease, ovarian clear cell carcinoma, ovarian mucinous carcinoma, ovarian low-grade serous carcinoma, and small cell ovarian carcinoma hypercalcemic type are the initial cancers classified by ESGO as rare gynaecological tumors. This work had already been started almost a decade ago by the Gynecologic Cancer InterGroup (GCIG), which included the Rare Tumor Working Group [5,6,7,14,15,16]. Nonetheless, the standardization of clinical/histopathological guidelines when defining a rare gynae tumor will be of paramount relevance both for the diagnosis itself, and for designing clinical trials for testing new therapies. Therefore, for both translational studies and clinical trials, pathology input is crucial to determine the diagnosis, using appropriate diagnostic criteria, including immunohistochemistry and, where appropriate, molecular testing.

### 2.2. Biobanks as Basis of Personalized Medicine

The development of new diagnostic, prognostic and treatment strategies will largely depend on our ability to study the molecular basis of RGT. At present, the great heterogeneity coming from inter-centre specimen handling (e.g., pre-analytic variations linked to its processing and storage) will conceivably result in an overall poor replicability/reliability of the findings obtained from multi-site-based studies, leading to a general inability of formulating robust conclusions. Therefore, the development of RGT dedicated biobanks, along with the definition of Standard Operating Procedures (SOAPs) will play a crucial role in collecting an adequate series of biospecimens with accompanying clinical data for personalized medicine [17,18]. Biobanks promise high quality biological samples for collaborative scientific research but have to face major challenges to achieve internationally recognised certification and/or accreditation. Challenges related to biobanking include quality management by adherence to International Standards, namely General Requirements for biobanking (ISO 20837: 2018) [19] and Quality management systems (ISO 9001:2015) [20], resolution of ethical and legal issues related to specimen access, on a national and international level, while ensuring adequate safeguards for participant privacy and data protection, as well as the development of strategies for long term biobank sustainability. Thus, harmonization of biobanking standards is very important in facilitating international multi-center collaborative studies with highly valuable outcomes to improve personalized treatments [21]. Several academic institutions and biotechnology companies committed to biobanking across the world (e.g., UK BioBank, Japan BioBank, FinnGen) are already implementing standardized conditions that allow an easy exchange of harmonized data and specimens with the final aim of increasing the sample size of the cohort analyzed (e.g., of particular relevance when dealing with rare conditions, such as gynae cancer) and the reliability/reproducibility of the findings. The engagement with EU-wide research infrastructures such as BioBanking and Biomolecular Research Infrastructure–European Research Infrastructure Consortium (BBMRI-ERIC) and other international consortia will strive to deliver advanced bio-banking resources to all contributors by providing for harmonisation of procedures with Standard Operating Procedures for the consenting, collection and pre-analytical processing of all data and samples [22]. This will facilitate the provision of multi-modal, harmonized data from the different branches of the -omic sciences (e.g., genomics, epigenomics, proteomics, etc.) [17].

Currently, the new field is that of imaging biobanks (IBs) is generating a large amount of data coming from cutting-edge imaging technologies that can be exploited by high-throughput computing to extract radiological features, useful to determine new non- or minimally invasive biomarkers. Imaging biobanks linked to biological samples and patients’ clinical information may be considered as a new frontier in biobanking. Similar considerations apply to the increasing generation of whole-slide images from histopathology slides, which are now used in some healthcare settings for routine pathology reporting. This could lead to the generation of multi-omics biobanks, where radiological and histopathological image data could be integrated with genomic, transcriptomic, proteomic, metabolomics, etc findings for an innovative and personalized approach to cancer treatment [17]. In this frame, we believe that the future of medical research should be closely linked to that of biobanking, which could offer tools to all researchers to overcome these challenges, facilitating development of novel diagnostic strategies and personalized treatments for RGT.

### 2.3. Assessing the Impact of Molecular Testing on RGT Treatment

Until the early 21st century, classification of human cancers, including gynecologic tumors, was mostly based on the tissue of origin or histological characteristics rather than well defined complex molecular signatures [23]. Subsequently, genetic defects, which basically determine the abnormal behavior of tumor cells, were analyzed more effectively using high throughput molecular methods, such as: Whole Genome or Whole Exome Sequencing (WGS/WES), Comparative Genomic Hybridization (CGH-array), gene expression profiling by probe-based microarray or by RNA-sequencing (RNA-Seq), array-based protein expression and methylation profiling, allowing more information to be obtained through a system-biology approach (genome, transcriptome, proteome) [24,25,26].

Such advances in our understanding of the molecular features underlying cancer biology have facilitated our ability to classify tumors based on their molecular signatures and the identification of “driver” alterations involved in cancer development and progression [27]. Indeed, this extensive molecular characterization is paving the way for a tailored, therapeutic precision medicine approach for each individual patient [28]. As the majority of individual molecular alterations identified so far do not have an ad-hoc FDA-or EMA-approved therapy, the need for larger studies employing high-throughput technologies for molecular testing, to better define the cancer-related aberration, is clearly warranted. However, there is still much to be learnt on the optimal timing of testing and incorporation into clinical practice [29].

Tumor recurrence after initial therapy is usually fatal in most cancers, including gynaecological. Now, when standard of care options fail, molecular testing such as next generation sequencing (NGS), Sanger sequencing and pyrosequencing, is increasingly utilized to identify targeted therapies for cancer treatments such as hormone therapies, pathway specific therapies, and immunotherapies. Molecular testing can identify opportunities for drugs approved by regulatory agencies as well as experimental therapeutics in clinical trials [29]. Thus, regarding RGT, it will be necessary to evaluate how the findings coming from the application of High-Throughput technologies (e.g., NGS, proteomics and other molecular testing) could lead to effective clinical actions, through the identification of predictive biomarkers useful both in disease prediction and in its clinical follow-up, by means of a pharmacogenomics approach.

### 2.4. Prospects of Omics-Based Molecular Approaches in RGT Diagnosis and Treatment

As mentioned above, in recent years, high-throughput technologies (genomics, transcriptomics, proteomics and metabolomics) (Table 2) have demonstrated enormous potential as unbiased, large-scale, biomarker discovery platforms [30]. Genomics provides information about the full set of genes within a cell, rather than focusing on individual genes, and holds a great promise to enhance the discovery of novel biomarkers leading to diagnostic tests [31,32].

Genomics has truly revolutionized clinical diagnostics. However, focusing on just the genome may not be enough to better elucidate disease mechanisms. To begin with, environmental factors may also play a role in disease causation. In addition, protein-coding sequences constitute only 1.5–2% of the human genome, whereas the majority of the genome is transcribed into non-coding (nc) RNAs, including microRNAs (miRNAs), long non-coding RNAs (long ncRNAs), small interfering RNAs (siRNAs), piwi-interacting RNAs (piRNAs), transfer ribonucleic acids (tRNAs), ribosomal ribonucleic acids (rRNAs), small nuclear RNAs (snRNAs), and small nucleolar RNAs (snoRNAs) [33].

Thus, these complexities necessitate use of multi-focal approaches that involve different omics techniques [34,35]. Moreover, epigenome should also be considered as a collection of personal and dynamic changes that may be involved in disease mechanisms. Epigenetic mechanisms (DNA methylation, histone modifications and nucleosome remodeling) have been shown to represent the prevalent carcinogenetic player [35]. Epigenetics involves the role of non-coding RNAs (including microRNAs), that by regulating gene expression of their target genes, they may lead to their inhibition and/or degradation [36].

Epigenetic silencing has been suggested as one of the major causes of gynecologic cancer, being able to inactivate multiple pathways such as cell cycle control, DNA repair, and apoptosis [36]. Epigenetic alterations have been recognized as useful tools for the development of novel biomarkers for diagnosis, prognosis, and monitoring of diseases, as well as may represent novel therapeutic targets [37,38].

The study of the transcriptome allows for the characterization of genetic expression at the RNA level. In contrast to DNA, RNA which is actively transcribed, reflects the diversity of cell types and their regulatory mechanisms [35]. Moreover, it is well established that cancer cells display aberrant transcriptional patterns underlying the pathogenic disruption at the basis of the various cancer related phenotypes. In addition to deregulated transcription, it has been shown that mutations at splicing-site sequences and/or affecting the spliceosome machinery lead to aberrant splicing in many cancers [39]. Thus, RNA sequencing allows the identification of differentially expressed genes, cancer specific transcript isoforms and has great potential in unraveling underlying molecular mechanisms, helping the search for disease-specific biomarkers.

Furthermore, single-cell RNA sequencing (scRNA-seq) provides exceptional depth at a single cell resolution, revealing distinct trajectories, identifying populations of complex and rare cell lineages that cannot be detected from pooled cells. Overall, novel transcriptomic techniques are likely to offer functional clues to tumor progression and immunotherapy response of patients. However, transcripts may not always truly reflect the functional phenotype of a cell because they are not their final genetic products. There is also limited correlation between mRNA levels and encoded proteins. Therefore proteomics has great potential to yield clinically relevant biomarkers, since the proteome better reflects the dynamic state of cells and tissues [35].

Metabolic reprogramming is one of the hallmarks of cancer and consequently, many metabolites detected in the blood can show altered levels in cancer patients. Metabolomics differs from nucleic acid-based-omics methods, because methods such as, liquid chromatography, mass-spectrometry (LC-MS/MS) and nuclear magnetic resonance spectroscopy (NMR), metabolites contained in a sample can be detected and quantified [34]. Metabolomics is based on the premise that any differences in metabolites reflect differences in biological processes, and therefore hifts in metabolite composition (such as the profile of free amino acids in plasma) and changes at the genetic level allow the screening of potential biomarker candidates or therapeutic targets. The recent advancesin metabolomics could be pivotal in early cancer detection [40]. So, this knowledge might lead to the development of new metabolomics-based screening tools for early detection of a malignancy. These findings can then be integrated with genomics, transcriptomics, proteomics and epigenomic data to accelerate cancer research and diagnostics [41].

On this basis, our future studies will aim to characterize RGT by high-throughput technologies (genomics, epigenomics, transcriptomics, proteomics, and metabolomics). The achievement of this goal will allow to improve not only the classification of tumors but also to identify new therapeutic targets and strategies for RGT.

### 2.5. Assessment of Circulating Tumor-Specific Markers as Predictive Biomarkers in RGT

The availability, quantity and quality of tumor tissue pose a substantial challenge to the clinical implementation of personalized medicine. The processing of formalin-fixed, paraffin-embedded fragments can alter nucleic acids, and thus can decrease test sensitivity or lead to false-positive mutation calls. Moreover, the use of archived tissue or biopsies collected at a single time point may not account for all the intra-tumoral heterogeneity in space or time. Acquisition of multiple tumor biopsies from the same patient to overcome this issue, is hindered by the need for undertaking invasive procedures that may put patients physically at risk, and also require a significant amount of resources [42]. So, there is an urgent need for less-invasive procedures and new tumor biomarkers to improve early diagnosis.

Tumor biomarkers are molecules that are produced by cancer or stromal cells around them, which can be measured in body fluids such as blood, urine, and peritoneal fluid during the diagnosis, screening, or treatment of cancer. An ideal tumor biomarker has to be be sensitive enough for early detection of small tumors while at the same time retaining the specificity of the identified cancer type [43]. To date, there is no known tumor biomarker carrying these features for any of the RGT.

An emerging field that may ameliorate this issue is the testing of circulating tumor-specific markers such as circulating tumor cells (CTCs), circulating tumor DNA (ctDNA), RNAs, particularly circulating free microRNAs (miRNA), proteins, metabolites, or exosomes, that are present in body fluids [42,44,45].

In this regard, liquid biopsies are easily accessible by means of minimally invasive procedures that can be repeated to provide a dynamic assessment of tumor-specific biomarkers. In addition, although most studies have focused on the detection and identification of blood biomarkers, urine samplescould also provide a promising resource for the screening of cancer patients [42,44,45]. Hence, our future research will aim to elucidate if liquid biopsies and/or urine could enable the development of routine screening tests, leading to early diagnosis, and reducing the poor survival rates associated with the later detection and treatment of RGT.

### 2.6. Modern Approaches to Improve the Diagnosis and Treatment of RGT

The first line of therapy for most gynecologic cancers includes surgery, which is preceeded or followed by chemotherapy and/or radiation [46]. In the majority of cases, these traditional therapies do not completely remove the malignant cells. Therapeutic targets and modern immunotherapy, including PDL1 inhibitors, anti-angiogenic drugs, and PARPi are showing promising results [47].

However, the main reason for the high mortality is tumor recurrence and subsequent metastasis caused by cancer stem cells (CSCs). These are a subpopulation of cells with the ability to undergo self-renewal and clonal evolution, and thus play a key role in drug resistance and tumor progression. CSCs have been identified in a number of solid tumors, including in several gynecologic malignancies (Table 3) [48,49,50,51,52,53,54,55,56,57,58,59]. Thus, new targeted strategies, possibly targeting CSCs, are urgently needed to minimize morbidity and mortality associated with RGT.

In several advanced cancers which are difficult to treat, theranostic approaches combining diagnostic imaging with therapy, have been shown to improve patient survival [60], and could also be applied to RGT. The theranostic concept relies on identifying appropriate molecular targets which are highly specific to cancer cells and, by using specific imaging techniques, their expression levels and distribution is assessed sothat it can be subsequently utilized for guiding appropriate therapy [60].

Advances in nanotechnology have led to next-generation nanotheranostics (NGNT) using multifunctional smart ‘all-in-one’ nanoparticles which combine diagnostic, targeting and therapeutic agents into one single biocompatible biodegradable carrier. This nanotechnology has paved the way for breakthroughs in early detection and treatment of cancer through efficient targeting of CSCs [61].

Optimum NGNT must abide to the following criteria: (1) Rapidly and selectively accumulate in specific target site avoiding deposition of the therapeutics in healthy tissues; (2) allow a maximum drug loading capacity; (3) ability to signal morphological and biochemical characteristics of its target; (4) confer smart controlled drug release; and (5) ability to be cleared from the body after finalizing their task or biodegraded into nontoxic by-products. The perfect design of these NGNT should include four main components: therapeutic biomedical payload, imaging agent, nanocarrier and targeting moieties attached to the carrier surface. Achieving all these challenges by NGNT fabrication will open up new avenues for breakthroughs in better management through early diagnosis and treatment of cancer through the efficient targeting of CSCs [61].

The gene editing approach is also currently investigated for its potential use in cancer therapy. The Clustered Regulatory Interspaced Short Palindromic Repeats (CRISPR) system consists of an RNA-guided nuclease Cas9, and the small guide RNA (sgRNA), an RNA molecule with a 20-nucleotide RNA sequence complementary to a specific target sequence in the genome. The sgRNA guides and activates the Cas9 nuclease, which makes a double-stranded break at the designated site, which is then repaired by cellular machinery. This repair can be guided to make a specific desired change in the genome, such as targeted insertion, deletion or correction of a specific DNA sequence, making it a potentially revolutionary tool for therapy [62]. Cancer therapy with the CRISPR-Cas9 technology is not focused on correcting/killing tumor cells, as the delivery and targeting of specific tissues is still an issue. The approach taken by many researchers is rather to modify the immune system of the individual, making their immune cells more responsive and more reactive, thus enabling them to recognize and destroy tumor cells [63]. A similar approach is taken by the therapeutic vaccines, which also primarily act as hyperactivators of the immune system. Even though there are still limitations to such therapies, clinical trials of therapeutic vaccines and CRISPR-Cas9 technology for ovarian cancer are under way [64]. Nowadays, RGT are frequently misdiagnosed or else diagnosed at an advanced-stage due to the lack of effective biomarkers. So, our future research will aim to identify, using high-throughput technologies, new molecular targets and to develop novel approaches for cancer targeting with an emphasis on detection of CSCs, thus, improving both diagnosis and treatment of RGTs.

## 3. Conclusions

Almost 50% of gynecologic cancers and up to 25% of cancer mortality is represented by rare cancers [65]. Mortality rates for RGT are still high due to the current lack of understanding of their pathophysiology. International collaborative efforts to investigate these RGT may lead to a better harmonization of practice with potentially more effective treatment [66]. Currently, many RGT are still being excluded from clinical trials or else included in trials studying other cancers from the same organ of origin, but from which these rare cancers might be very different on a molecular level.

So, the future challenge will be to accurately diagnose patients, followed by designing clinical trials for these RGT. Investigator-led international trials with the support of the pharmaceutical industry will enable to further the current knowledge base.

We also believe that overcoming the challenges described here, may lead to the following important outcomes: (1) Advancement of the state-of-the-art in the field of RGT; (2) development of new less- or non-invasive diagnostic methods for an earlier diagnosis, as well as improving RGT treatment; (3) an increase in the application of nanotechnology both in diagnosis and therapy; and (4) development of prospective databases with biobanking.

Overall, we expect that, international collaborations such as GYNOCARE (COST Action CA18117), will allow a significant step forward in improving the quality of RGT research, and thus will result in improving clinical care through personalized management of those women suffering from RGT.

## Figures and Tables

**Table 1:** Rare gynecological tumors *.

Site	Morphology	Malignancy
Vulva-Vagina	Epithelial	Paget’s disease of the vulva
		Adenocarcinoma
		Other carcinomas
		Skin adnexal carcinoma
	Germ Cell	Yolk sac tumor and other types
Uterine cervix	Epithelial	Adenocarcinoma
		Carcinosarcoma
	Mixed	Adenosarcoma
Uterine corpus	Epithelial	Carcinosarcoma
	Mixed	Adenosarcoma
	Germ Cell	Yolk sac tumor and other types
Fallopian tube	Epithelial	Adenocarcinoma
	Mixed	Adenosarcoma
Ovary	Epithelial	Mucinous adenocarcinoma
		Clear cell adenocarcinoma
		Low-grade serous carcinoma
		Other carcinomas
	Sex cord-stromal	Adult granulosa cell tumor
		Juvenile granulosa cell tumor
		Sertoli-Leydig cell tumor
		Steroid cell tumor
	Mixed	Adenosarcoma
	Germ Cell	Dysgerminoma/Seminoma
		Yolk sac tumor
		Mixed germ cell tumor
		Embryonal carcinoma
		Choriocarcinoma, NOS
		Immature teratoma
		Gonadoblastoma
All sites	Mesenchymal	Sarcomas eg leiomyosarcoma
	Mesonephric (Wolffian system)	Wolffian tumor, Mesonephric carcinoma
	Neuroendocrine neoplasia	Neuroendocrine carcinomas and mixed neuroendocrine -non neuroendocrine carcinomas
	Haematolymphoid	Primary haematolymphoid tumors
Gestational trophoblastic disease	Trophoblastic	Choriocarcinoma
		Epithelioid trophoblastic tumor
		Placental site trophoblastic tumor

* Adapted from WHO classification of tumors Editoral Board. Female Genital Tumors. Lyon (France) IARC 2020 (WHO classification of tumors series, 5th ed.; Volume 4). https://publications.iarc.fr/592.

**Table 2:** Some High-throughput technologies and Features.

Objective	Type of Analysis	Feature
Genomic	Whole-Genome Sequencing (WGS)	To detect DNA mutations and structural variations by sequencing the whole genome
	Whole-Exome Sequencing (WES)	To detect DNA mutations by sequencing the whole exon region
Epigenomic	Bisulfite sequencing	To analyze genomic DNA methylation
	ChIP-seq ^a^	To detect the targets of transcription factors or analysis of histone modifications
	miRNA Sequencing	To analyze microRNA (miRNA) expression profiles
Transcriptomic	RNA sequencing	Used for analysis of gene expression or detection of fusion genes and splice variants
Proteomic	Microarray	To detect only known proteins
	SELDI-TOF MS ^b^	To perform omni-comprehensive protein profiling
Metabolomic	^c^ LC-MS plus NMR	To separate and detect metabolites

^a^ ChIP: Chromatin immunoprecipitation; ^b^ SELDI-TOF MS: Surface-enhanced laser desorption/ionization-time of flight mass spectrometry; ^c^ LC-MS plus NMR: liquid chromatography and mass spectrometry plus nuclear magnetic resonance.

**Table 3:** Cancer Stem Cells reported in gynecologic cancers [46,47,48,49,50,51,52,53,54,55,56,57].

Gynecologic Cancer	CSC Marker(s)
Cervical cancer	* SP; or ALDH^high^
Uterine cancer	* SP; or CD133^+^
Ovarian cancer	CD44^+^CD117^+^; CD44^+^CD24^−^; ALDH^high^CD133^+^; or CD24^+^
Vulvar cancer	CD133^+^

* SP: Side-population.

## Data Availability

This concept paper does not report any new data.

## Abbreviations

ALDH	aldehyde dehydrogenase
BBMRI-ERIC	BioBanking and Biomolecular Research Infrastructure–European Research Infrastructure Consortium
ChIP	Chromatin immunoprecipitation
CRISPR	Clustered Regulatory Interspaced Short Palindromic Repeats
CSC	cancer stem cell
CTC	circulating tumor cell
ctDNA	circulating tumor DNA
EMA	European Medicines Agency
FDA	Food and Drug Administration
ERNs	European Reference Networks
JARC	Joint Action on Rare Cancers
LC-MS	liquid chromatography and mass spectrometry
miRNA	micro-RNA
NGS	next generation sequencing
NMR	nuclear magnetic resonance
RAC	rare adult cancers
RGT	rare gynecological tumor
scRNA-seq	single-cell RNA sequencing
SELDI-TOF MS	Surface-enhanced laser desorption/ionization-time of flight mass spectrometry
SOAPs	Standard Operating Procedures
SP	Side-population
